# Idiopathic Giant Hepatic Artery Pseudoaneurysm

**DOI:** 10.1155/2017/4658065

**Published:** 2017-10-17

**Authors:** Ahmed Abdelbaki, Neeraj Bhatt, Nishant Gupta, Shuo Li, Shady Abdelbaki, Yogesh Kumar

**Affiliations:** ^1^Department of Radiology, Yale New Haven Health, Bridgeport Hospital, 267 Grant St, Bridgeport, CT 06610, USA; ^2^Department of Radiology, St Vincent's Medical Center, 2800 Main St, Bridgeport, CT 06606, USA; ^3^Department of Medicine, Yale New Haven Health, Bridgeport Hospital, 267 Grant St, Bridgeport, CT 06610, USA; ^4^Department of Radiology, Columbia University at Bassett Healthcare, 1 Atwell Rd, Cooperstown, NY 1332, USA

## Abstract

Hepatic artery pseudoaneurysm (HAP) incidence is rising due to more common use of endoscopic and percutaneous hepatic interventions. HAP is potentially fatal, as it could lead to sudden life-threatening hemorrhage. HAP can be intrahepatic or extrahepatic. On computed tomography angiogram (CTA) and magnetic resonance angiogram (MRA), HAP follows blood pool on multiphasic examination, with brisk arterial enhancement that washes out, similar to the abdominal aorta on later phases. We present a case of idiopathic giant HAP in an 82-year-old male. Currently, angioembolization is replacing surgery as the initial modality of choice for management of this condition.

## 1. Introduction

Pseudoaneurysms occur when there is a breach in the vessel wall with blood leaking through the wall but contained by the adventitia or surrounding soft tissue [1]. Visceral artery aneurysms are uncommon, with an incidence of 0.1% to 10.0% in autopsy series [2]. HAP is a rare complication of liver trauma and liver transplant [3, 4]. Hepatic artery aneurysms incidence is estimated at 0.002%, and approximately 50% of hepatic artery aneurysms are pseudoaneurysms [5]. The incidence of HAP is on the rise due to more frequent use of percutaneous and endoscopic interventional procedures. HAP could be asymptomatic. The use of anticoagulation therapy is associated with increased risk for developing femoral artery pseudoaneurysms [6]. However, upon review of the literature, there is no association between anticoagulation therapy and HAP [4, 5, 7, 8]. HAP may present with nonspecific symptoms including hemobilia, falling hemoglobin levels gastrointestinal bleeding, or hemoperitoneum [9].

## 2. Case Report

**Figure 1:**
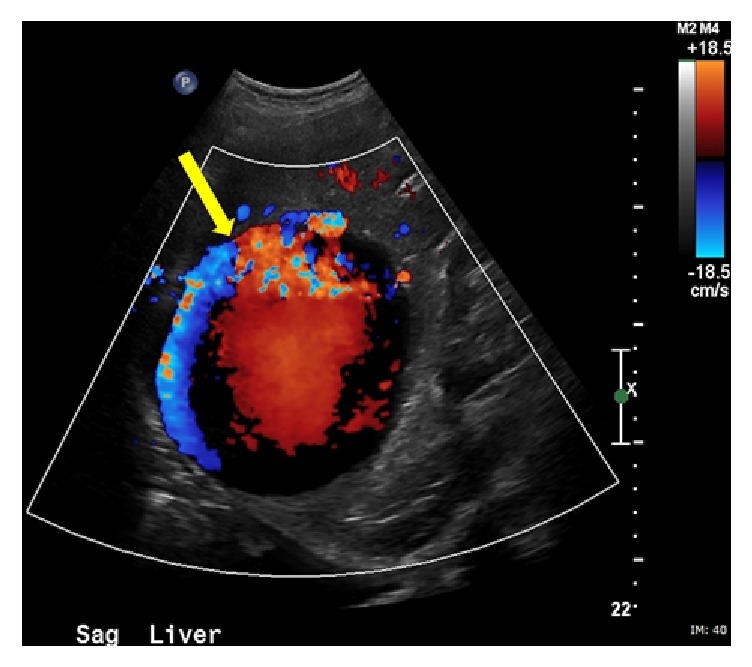
Color Doppler ultrasound demonstrating bidirectional blood flow within an intrahepatic pseudoaneurysm “Ying-Yang sign” (yellow arrow).

**Figure 2:**
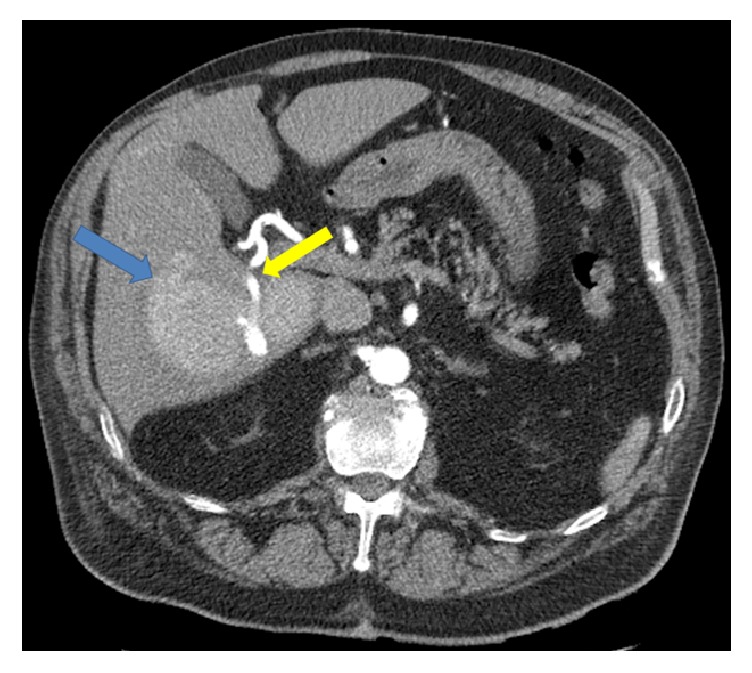
Axial plane CT angiography of the abdomen early arterial phase demonstrates contrast filling of an intrahepatic pseudoaneurysm (blue arrow) by a prominent right hepatic artery (yellow arrow).

**Figure 3:**
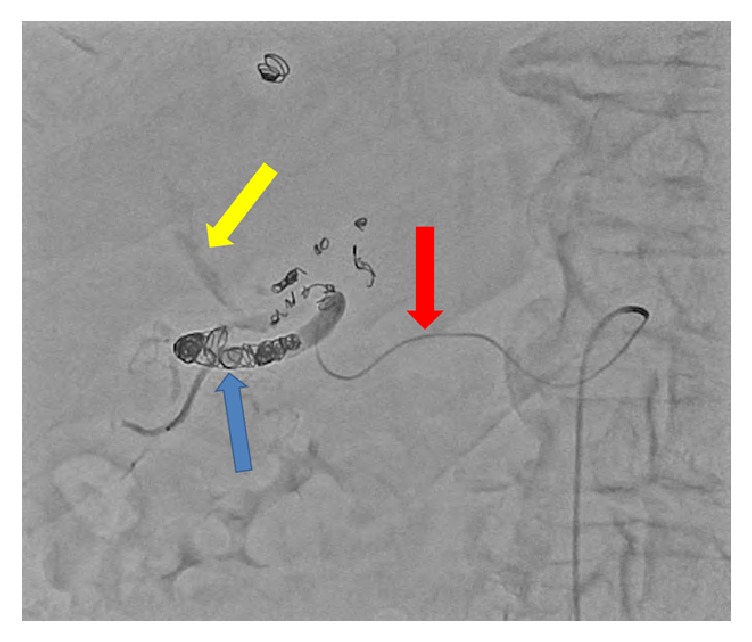
Anterior projection digital subtraction angiography demonstrating selective cannulation of right hepatic artery (red arrow). Multiple embolization coils (Tornado 0.08 embolization microcoils and interlocking 2D helical 8 × 20 coils) are filling the right hepatic artery lumen (blue arrow). Few displaced coils and mild residual contrast blush is noted within the aneurysm sac (yellow arrow).

**Figure 4:**
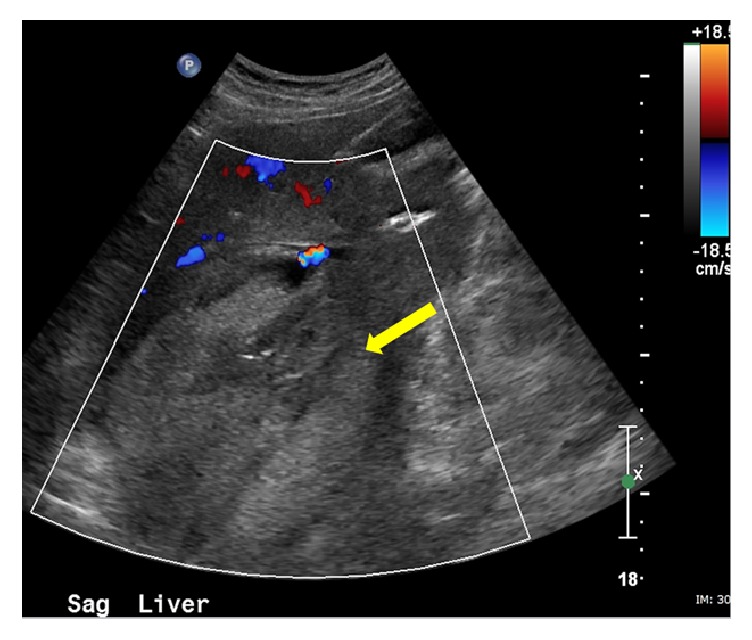
Postembolization color Doppler ultrasound demonstrating echogenic thrombus occupying the aneurysm sac with near total absence of color flow (yellow arrow).

**Figure 5:**
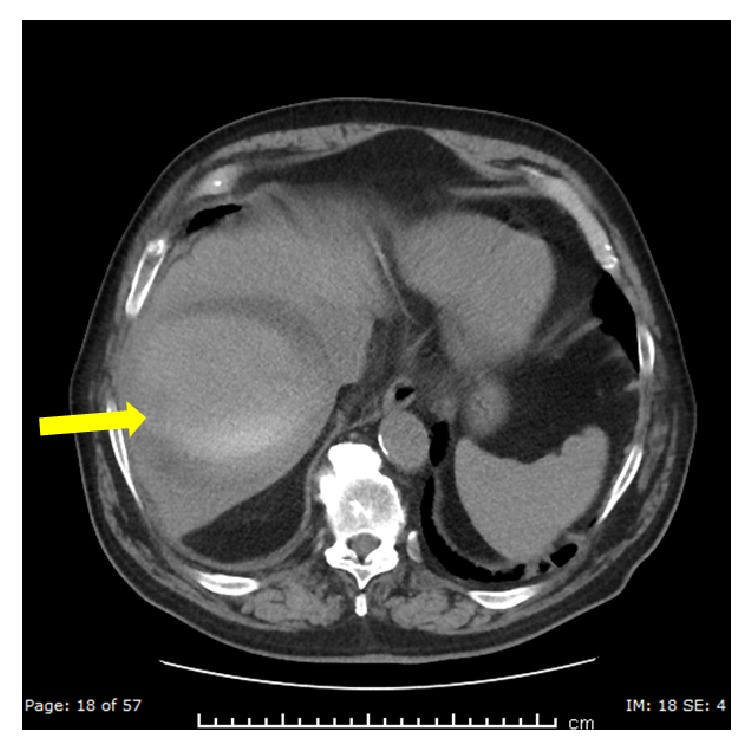
Axial plane CT of the abdomen without contrast done 1 week after the embolization demonstrates a large collection replacing most of the right hepatic lobe. There is central increased attenuation with layering (yellow arrow) consistent with retracting clot.

**Figure 6:**
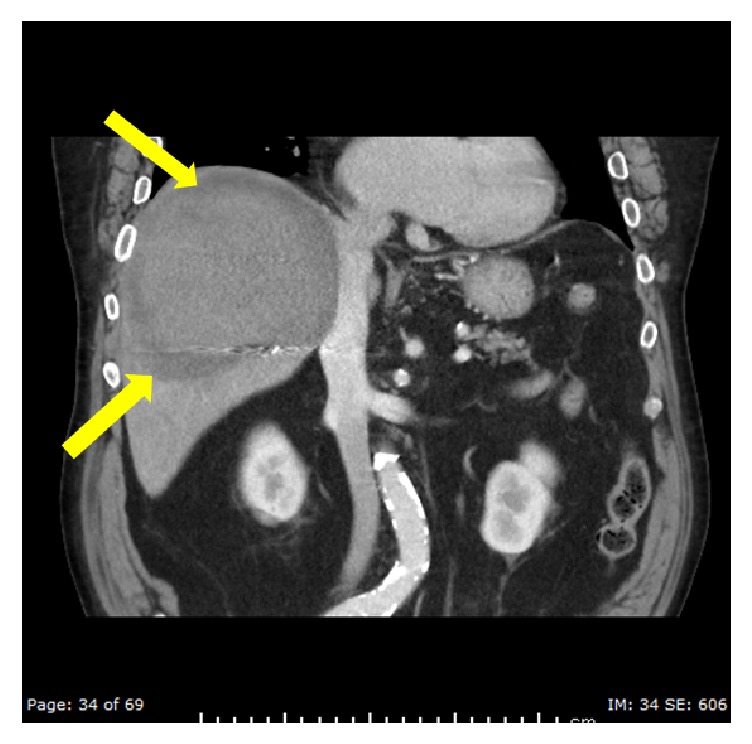
Coronal plane CT of the abdomen after embolization with administration of intravenous contrast in portal phase done 1 week after the embolization, demonstrating the collection with no active extravasation. There is a peripheral rim of low attenuation likely related to infracted hepatic parenchyma (yellow arrows).

The patient is an 82-year-old male with history of atrial fibrillation, coronary artery disease, and hypertension. He was on anticoagulation. He presented to the emergency department with shortness of breath for which he underwent a CT of the chest without contrast. A large 14 cm hypodense liver mass was incidentally detected. Further evaluation with dedicated liver protocol magnetic resonance imaging (MRI) revealed a large mass in the right hepatic lobe showing intense and homogeneous arterial phase enhancement that persisted on delayed images. The mass followed the signal intensity of aorta throughout different phases of contrast enhancement. On abdominal duplex US, brisk internal color flow was seen as well as bidirectional blood flow “Ying-Yang sign” (Figure 1). Confirmation with CTA of the abdomen revealed a large arterial phase hyperenhancing liver mass. Immediately adjacent to this mass was an enlarged branch of the right hepatic artery from which a contrast jet was extending into the mass, producing a vascular blush (Figure 2). These findings were consistent with giant HAP. Given the large size of the pseudoaneurysm, open vascular surgical interventions were deferred due to technical difficulties and associated increased morbidity and mortality [10]. Interventional radiology was consulted for endovascular management. Endovascular management is associated with lower morbidity and shorter period of hospitalization. According to Tessier et al., endovascular management is associated with 86% success rate [10]. Recurrence of HAP is mainly due to vascular recanalization; this is usually treated with repeat embolization or surgical resection [11]. Initial angiogram demonstrated a large pseudoaneurysm getting predominant supply from an enlarged branch of the posterior segment of the right hepatic artery. In addition, there were multiple smaller feeders arising from right hepatic, left hepatic, gastroduodenal, and pancreaticoduodenal arteries. The dominant feeder from right hepatic artery was coiled using multiple embolization coils (Tornado 0.08 embolization microcoils and interlocking 2D helical 8 × 20 coils). Subsequent arteriogram demonstrated additional small feeding branches from both the right and left hepatic arteries, which were also embolized (Figure 3). Follow-up abdominal ultrasound performed one day after embolization demonstrated near complete thrombosis of the pseudoaneurysm with a large heterogeneous clot in the lumen of the pseudoaneurysm sac (Figure 4). Repeat CTA of the abdomen done 1 week after embolization demonstrated a large organizing hematoma in the right hepatic lobe with no vascular blush (Figures 5 and 6).

## 3. Discussion

HAP is potentially fatal, as it could lead to sudden life-threatening hemorrhage. HAP can be intrahepatic or extrahepatic. Ultrasound demonstrates a cystic structure along the course of hepatic artery with brisk internal color flow, to-and-fro pattern and Ying-Yang sign [12]. CTA of the abdomen and pelvis can be very helpful in cases of visceral aneurysms and pseudoaneurysms [13]. The pseudoaneurysm follows blood pool on multiphasic contrast examination, with brisk arterial enhancement that washes out, similar to the abdominal aorta on later phases [1, 12]. The incidence of hepatic artery aneurysm rupture has been reported to be within 21%–80% [5, 14, 15]. Mortality from hepatic artery aneurysm rupture is 21%–43% [14, 15]. Hepatic artery pseudoaneurysms have increased risk of rupture [16]; however, the exact incidence is unknown. Management of HAP is a big challenge. It depends on severity, location, and underlying etiology. In mycotic pseudoaneurysms, surgical resection is the routine treatment, because endovascular material may serve as an infectious nidus. Intravascular occlusion can be beneficial as a temporizing measure to stabilize patients with active bleeding prior to surgery. Percutaneous thrombin injection is widely used to treat peripheral pseudoaneurysms [16]. Percutaneous thrombin injection has also been described for the management of visceral pseudoaneurysms [17]. Coil embolization is one of the cornerstones in treating the intrahepatic pseudoaneurysms [8]. Angioembolization results in rapid bleeding control, lower transfusion requirement, and shorter hospital stay [7]. Currently, angioembolization is replacing surgery as the initial modality of choice [10].

## 4. Conclusion

HAP incidence is rising. On CTA and MRA, HAP follows blood pool on multiphasic examination, with brisk arterial enhancement that washes out, similar to the abdominal aorta on later phases. Currently, angioembolization is replacing surgery as the initial modality of choice.
